# Clonal Dynamics of Nasal *Staphylococcus aureus* and *Staphylococcus pseudintermedius* in Dog-Owning Household Members. Detection of MSSA ST^398^

**DOI:** 10.1371/journal.pone.0069337

**Published:** 2013-07-09

**Authors:** Elena Gómez-Sanz, Carmen Torres, Sara Ceballos, Carmen Lozano, Myriam Zarazaga

**Affiliations:** Área Bioquímica y Biología Molecular, Universidad de La Rioja, Logroño, Spain; Universitätsklinikum Hamburg-Eppendorf, Germany

## Abstract

The objective of this study was to investigate the dynamics of nasal carriage by *Staphylococcus aureus* (SA) and *Staphylococcus pseudintermedius* (SP) among healthy dog-owning household members involved in 7 previous index cases of suspected anthropozoonotic (n = 4) and zoonotic (n = 3) interspecies transmission [4 direct cases, identical SA (n = 3) or SP (n = 1) in owner and dog; three indirect, SP in owner (n = 2) or SA in dog (n = 1)]. Co-carriage with methicillin-resistant coagulase-negative staphylococci (MRCoNS) was also evaluated. Sixteen owners and 10 dogs were sampled once every three months for one year. In total, 50 SA and 31 SP were analysed by MLST, and SA also by *spa* typing. All isolates were subjected to *Apa*I/*Sma*I-PFGE and antimicrobial resistance and virulence profiles were determined. All index owners were persistent SA carriers in all direct-anthropozoonotic transmission cases, while only one dog was persistent SA carrier. Owner and dog exhibited a persistent SP carriage status in the direct-zoonotic transmission case. SP was maintained in the index human over time in one indirect-zoonotic transmission case. Only one SP was methicillin-resistant. SA belonged to genetic backgrounds of MRSA pandemic clones: CC45, CC121, CC30, CC5 and CC398. Three individuals carried a MSSA t1451-ST398 clone with the *erm*(T)-*cadD/cadX* resistance genes. SA or SP were persistently detected in the nasal cavity of 7 (43.8%) and 2 (12.5%) owners, and in one and 2 dogs, respectively. SA was recovered as the single species in 10 owners and in one dog; SP in 3 owners and 4 dogs; and both bacterial species in one owner and 4 dogs. Co-carriage of SA or SP with MRCoNS isolates was common (30.7%). This is the first study on the dynamics of nasal carriage of SA and SP in healthy pet-owning household members. Dog-contact may play a role in the staphylococcal species distribution of in-contact individuals.

## Introduction

*Staphylococcus aureus* (SA) and *Staphylococcus pseudintermedius* (SP) form part of the normal microbiota of the nares, skin and mucous of humans and dogs, but they are also opportunistic pathogens [1], [2]. SA can be also found in dogs at moderate rates (<20%) with a suggested anthropozoonotic origin, although the direction of transmission has not yet been elucidated [3]–[7]. Humans can carry SP at very low frequencies [8], [9], while higher rates are generally detected among individuals with regular dog contact (≤4.5%) [2], [3], [7], [10], [11].

Analysis of the colonization dynamics over time is essential to address the real carriage status of a positive individual and for a better understanding of the interaction among staphylococci in the distinct hosts. However, very limited data do exist on the longitudinal carriage of SP in humans in contact to dogs [12]–[14], and none among healthy individuals. A couple of recent studies on the carriage dynamics of methicillin-resistant SP (MRSP) in dogs and in-contact humans reported humans to be rarely colonized by MRSP for prolonged periods of time [13], [14]. Various reports have focused on the SA colonization dynamics in humans, including specific populations, such as homeless people, drug users, healthy infants, health care workers, subjects with staphylococcal (previous) infection [15]–[22] or, more recently, in individuals in contact to livestock [23], [24]. Nonetheless, to our knowledge, no longitudinal studies on SA from pet-owning humans and their pets have been reported to date.

On the other hand, humans can be carriers of methicillin-resistant coagulase negative staphylococci (MRCoNS), which normally harbor multiple antimicrobial resistance genes [25]–[27]. Although these bacteria are considered reservoirs for the exchange of genetic material between different staphylococcal species [28]–[31], co-carriage of SA or SP and MRCoNS in humans and in-contact pets has not been previously analyzed.

In this study, we investigated the dynamics of SA and SP nasal carriage of dog-owning household members with previous suspected cases of SA or SP interspecies transmission [7] to evaluate the individual carriage status and to estimate the direction of bacterial transmission. In addition, we sought to assess the co-carriage of these bacterial species and MRCoNS, to appraise the possible risk of gene transfer between those microorganisms. Our results suggest that pet-owning may play a relevant role in the staphylococcal species distribution of in-contact individuals.

## Materials and Methods

### Description of Investigated Households and Index Cases

**Figure 1:**
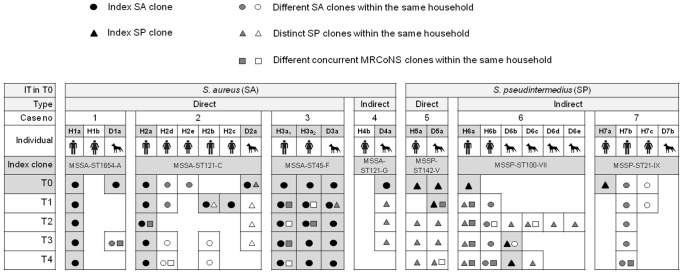
Schematic representation of the dynamics of carriage of the seven households investigated. MRCoNS, methicillin-resistant coagulase negative staphylococci. IT, bacterial species responsible for interspecies transmission. Type, type of interspecies transmission (Direct, Indirect). Case no, number of the households investigated in this study. Individuals are named H (for human) or D (dog) followed by the case number (1 to 7) and a lower-case letter to differentiate subjects per household; if necessary, a lower script number was added. Nomenclature of individuals involved in index cases (T0) is displayed with gray background. T0 to T4, the different samplings. Index clone, *S. aureus* or *S. pseudintermedius* clones responsible for interspecies transmission in index cases (T0).

Healthy owners and dogs coming from 7 households with previous description of suspected SA (4 cases, 1–4) or SP (3 cases, 5–7) interspecies transmission, recently detected in a study performed at the University of La Rioja (Spain) [7], were further investigated in the present study. Index cases were defined as cases in which SA or SP interspecies transmission is presumed to have occurred in original sampling (named T0), between at least one owner and one pet. All individuals tested were healthy and none has suffered from previous SP or SA infection. Direct interspecies transmission was considered when both, owner and dog, exhibited an identical SA or SP clone. Indirect interspecies transmission was defined as the presence of SA in dog but not in human, or SP in owner but not in dog. Figure 1 shows the composition of the residences, the bacterial species responsible and the different types of suspected interspecies transmission.

In the former study, household number assigned to the different index cases was as follows (case number in the present study): household no.1 (case 1), household 4 (case 2), household 2 (case 3), household 23 (case 4), household 5 (case 5), household 12 (case 6) and household 11 (case 7) [7]. Clone nomenclature used in this study is indicated as bacterial species and methicillin resistance pattern+Multilocus Sequence Type (MLST)+Pulsed-Field-Gel-Electrophoresis (PFGE) pattern. Index clones, defined as clones detected in index cases are shown in Figure 1 [7]. Several further isolates, not involved in the interspecies transmission index cases, were detected in T0 in the coexisting individuals (Figure 1). In total, 17 isolates (12 SA and 5 SP) obtained in T0 from the 7 households were included in the current study.

None of individuals tested presented occupational exposure to pets or the healthcare system. Both owners from cases 1, 3 and 6 were couples (range of age 27–30) while those from cases 2 and 7 were mother and/or grandmother (50–75 years old) plus 2 or 3 adults (son, daughter) (22–28). Owners from cases 4 and 5 (aged 27–35) lived alone with their dog. Individuals from cases 6 and 7 lived in rural areas.

### Study Design

In addition to 7 owners and 5 dogs involved in T0 index cases (named with the letter “a” and gray shaded in Figure 1), all coexisting individuals were also investigated (9 owners and 5 dogs) (Figure 1). Nasal samples from the anterior nares of 16 owners and 10 dogs were studied once every 3 months for one year (5 sampling times, T0 to T4) with a total of 130 samples analyzed. Sixty-four samples were recovered from owners and 40 samples from dogs during T1–T4 and completely characterized in this study. In addition, 26 samples from T0 from the former study were considered. Nasal co-carriage of SA and/or SP with MRCoNS was also investigated. All persons completed a written informed consent and agreed that their dog/s were also included. Sampling was approved by the Medical Ethical Committee of La Rioja (Permit Number: METC 09-399/C). Sterile swabs were provided to the owners in addition to detailed description of the sampling procedures. The owners swabbed themselves and the nares of their dogs. Nasal swabs were collected from the household owner within 24–48 h after sampling and they were immediately processed or eventually stored at −20°C until further analysis.

### Definition of the Carriage Status and Interspecies Transmission Dynamics

Subjects positive for SA or SP in at least four of the five samplings (including T0) were considered persistent carriers; those positive in two or three samplings were defined as intermittent carriers; individuals positive in a single sampling were reported sporadic carriers; and those negative throughout the study were defined as non carriers. It is important to remark the possibility that individuals who carry both bacterial species exhibited different carriage status per bacterial species. Dynamics of the interspecies transmission cases over time was defined likewise (persistent, intermittent, and sporadic).

### Isolation and Identification of SA, SP and MRCoNS Isolates

Samples were inoculated in Brain-Heart-Infusion broth (BHI, Difco) supplemented with 6.5% NaCl and incubated at 37°C for 24 h. One-hundred microliters were seeded on Oxacillin-Resistant-Staphylococcal-Agar-Base (ORSAB; OXOID) plates supplemented with 2 mg/L of oxacillin for the isolation of MR staphylococcal isolates, either SA, SP or CoNS. Seventy microliters were inoculated in parallel on Manitol-Salt-Agar (MSA; BD) plates for the isolation of SA and SP. Plates were incubated at 35°C for 24–48 h. All colonies with different morphology were subcultured on BHI agar (Difco) and further studied. Preliminary identification of isolates was based on colony morphology, Gram staining, and catalase and DNase activities. A species-specific multiplex PCR was performed to identify SA and *S. intermedius*/SP isolates [32]. Discrimination between *S. intermedius* and SP was conducted by restriction fragment length polymorphism of the *pta* gene [33]. The presence of *mec*A gene was investigated by PCR in all isolates [34]. Identification of MRCoNS was performed by amplification and sequencing of the *sodA* gene in all *mec*A positive CoNS isolates [35]. Only one MRCoNS isolate per individual was further characterized when isolates belonged to the same bacterial species.

### Analysis of the Clonal Relatedness of Isolates

The genetic relatedness of all isolates obtained (SA, SP and MRCoNS) was investigated by PFGE of total DNA restricted with *Sma*I or *Apa*I macrorestriction enzymes as previously described [36]. For SA and MRCoNS isolates, PFGE running conditions were those recommended by the HARMONY protocol [36]. SA isolates non-typeable with *Sma*I were subjected to *Apa*I-PFGE and run for 20 h at 6 V/cm using pulsed time ramping from 2 to 5 s [37]. *Sma*I-digested plugs obtained from SP isolates were run for 24 h at 5.6 V/cm using pulse times from 2 to 5 s [38]. Isolates were considered a unique clone when they showed up to three bands difference in PFGE band patterns and subclones when PFGE band patterns differed between 1–3 bands [39]. The different SA patterns were distinguished by capital letters, major SP patterns by Roman numbers and MRCoNS by Arabic numbers. Subclones were indicated with the major lettering type followed by a lower case letter.

### Molecular Typing of SA and SP Isolates

All SA isolates were subjected to *spa* typing as previously described [40] and sequences were analyzed using Ridom Staph-Type software version 2.0.21 (Ridom GmbH). The last recovered isolate of each novel clone per household was selected as representative strain for in-depth molecular characterization. MLST was performed as recommended (http://www.mlst.net) on representative SA isolate. MLST was determined in all SP isolates; for this, five housekeeping genes were amplified and sequenced (*pta*, *cpn60*, *tuf*, 16S rRNA and *agrD*) followed by assignment of alleles by comparison to those present in the NCBI nucleotide database and using a key table for MLST typing of *S. intermedius* group (SIG) isolates [38].

### Antimicrobial Resistance Profile of Isolates

Susceptibility testing to 17 antimicrobial agents was performed in all isolates obtained by agar disk-diffusion method [41]. Antimicrobials tested were as follows (µg/disk): penicillin (10U), oxacillin (1), cefoxitin (30) erythromycin (15), clindamycin (2), gentamicin (10), kanamycin (30), streptomycin (10U), tobramycin (10), tetracycline (30), trimethoprim-sulfamethoxazole (1.25+23.75), chloramphenicol (30), ciprofloxacin (5), mupirocin (200), fusidic acid (10), vancomycin (30), and linezolid (30). Methods and breakpoints followed for streptomycin and fusidic acid were those recommended by the Société Française de Microbiologie (http://www.sfm-microbiologie.org). Inducible or constitutive clindamycin resistance was determined by the double-disk diffusion test (D-test) [41].

Presence of 32 antimicrobial resistance genes was investigated by PCR in all representative SA and SP strains and in all MRCoNS isolates [34], [42]. Antimicrobial resistance genes tested were as follows: *mec*A, *blaZ*, *tet*(K), *tet*(M), *tet*(L), *tet*(O), *erm*(A), *erm*(B), *erm*(C), *erm*(T), *mph*(C), *msr*(A), *msr*(B), *aacA-aphD*, *aphA3*, *aadE*, *aadD*, *aadA*, *str*, *sat4*, *dfr*(A), *dfr*(D), *dfr*(G), *dfrK*, *cat_pC221_*, *cat_pC194_*, *cat_pC223_*, *fexA*, *cfr*, *mupA*, *fusB* and *fusC*.

### Detection of Virulence Genes

Presence of the Panton Valentine leukocidin (PVL) genes (*lukS/F-PV*) was determined in all isolates by PCR [7]. The leukocidins *lukE/D* and *lukM*, exfoliatins *eta* and *etb*, the toxic-shock syndrome toxin gene *tst* and 18 enterotoxin genes were likewise investigated in all representative SA strains [7]. These isolates were additionally tested by PCR for the presence of the human-specific immune evasion cluster (IEC) genes encoding the chemotaxis inhibitory protein (*chp*), staphylococcal complement inhibitor (*scn*) and staphylokinase (*sak*), enclosed within prophage φ3 [43].

The presence of leukocidin *lukS/F-I*, exfoliatins *siet*, *expA* and *expB,* 18 enterotoxin genes and the species specific *sec_canine_* and *se-int* genes were also investigated by PCR in all representative SP strains [7].

## Results

### SA Isolates and Clones Recovered and SA Population Structure

All isolates recovered from the 7 households are schematically represented in Figure 1.

Thirty-two SA isolates were obtained from 16 owners and 6 isolates were recovered from 10 dogs. The 12 SA isolates obtained in T0 were also considered [7]. In total, 50 SA isolates were recovered from the 7 cases.

Based on the PFGE band profile of the 50 SA isolates, 10 SA clones were detected (Table 1). Ten different *spa* types (Table 2) and 7 STs (ST1654, ST121, ST5, ST398, ST45, ST30, and a new ST registered as ST2619) were observed. Clone MSSA-ST45-F in owner H3a_1_, exhibited 3 different but related *spa* types along the sample year (T0→t073; T1→t116; T2→t026; T3-4→t073) (Table 2).

**Table 1:** Number of isolates detected and molecular characterization of SA and SP analyzed in this study.

	Clone^a^		Isolation data											
Case	SA	SP	Individual	T0	T1	T2	T3	T4	No isolates	Representative isolate	Resistance genes detected^b^	Relevant virulence genes detected^b^		
1	MSSA-ST1654-A		H1a	+	+	+	+	+	6	C3494	*blaZ*	[*seg, sei, sem, sen, seu*], (*chp*, *sak*)		
			D1a	+										
	MSSA-ST2619-B		D1a				+		1	C4897	*blaZ*	*lukED, etb*, (*seg, sei, sem, sen*), *sec*, (*scn*, *sak*)		
2	MSSA-ST121-C		H2a	+	+	+	+	+	8	C3931	*blaZ*, *tet*(K)	*lukED*, *etb*, [*seg, sei, sem, sen*], *sak*		
			D2a	+										
			H2b		+									
			H2c		+									
		MRSP-ST71-I	D2a	+					1	C3930	*blaZ*, *mec*A, *tet*(K), [*erm*(B), *aphA3*, *sat4*, *aadE*], *aacA/aphD*, *dfr*(G)	*lukS/F-I*, *siet*, *se-int*		
	MSSA-ST5-D		H2d	+	+				3	C3935	*blaZ*	*lukED*, [*seg, sei, sem, sen, seo, seu*], (*chp, sak*)		
			H2e	+										
		MSSP-ST77-II	D2a		+	+	+		4	C5102	*blaZ*, *tet*(K), *tet*(M), *erm*(A), *erm*(B), *aacA/aphD,* [*aphA3-sat4-aadE*], *cat_pC221_*	*lukS/F-I*, *siet*, *se-int*		
			H2c		+									
	MSSA-ST398-E		H2c				+	+	4	C5587	*blaZ*, (*erm*(T)^c^, *cadDX*)	*sec*, (*scn, chp*)		
			H2d				+	+						
3	MSSA-ST45-F		H3a_1_	+	+	+	+	+	15	C2919	*blaZ*	[*seg, sei, sem, sen, seo*], (*scn, chp, sak*)		
			H3a_2_	+	+	+	+	+						
			D3a	+	+	+	+	+						
		MSSP-ST6-III	D3a		+				1	C3953	*blaZ*	*lukS/F-I*, *siet*, *se-int*		
4	MSSA-ST121-G		D4a	+					1	C2727	-	*lukED*		
		MSSP-ST42-IV	D4a		+	+	+		3	C5552	*blaZ*	*lukS/F-I*, *siet*, *se-int*		
5		MSSP-ST142-V	H5a	+					3	C2915	*blaZ*, *tet*(K), *tet*(M), *erm*(B)^c^, [*aphA3*, *sat4*, *aadE*], *dfr*(G), *cat_pC221_*	*lukS/F-I*, *siet*, *se-int*		
			D5a	+	+									
		MSSP-ST185-VI	H5a			+	+	+	6	C5578	*blaZ*	*lukS/F-I*, *siet*, *se-int*		
			D5a			+	+	+						
6		MSSP-ST100-VII	H6a	+					3	C3469	*blaZ*	*lukS/F-I*, *siet*, *se-int, expA*		
			D6b				+	+						
		MSSP-ST70-VIII	H6a		+	+	+	+	9	C5562	*blaZ*	*lukS/F-I*, *siet*, *se-int, expA,* (*sec_canine_, sel*), *sea*		
			D6b			+								
			D6c			+		+						
			D6d			+								
			D6e			+								
	MSSA-ST30-H		H6b		+	+	+	+	4	C5559	*blaZ*	(*seg, sei, sem, sen, seo,seu*), *sak*		
	MSSA-ST398-E		D6b				+		1	C5086	*blaZ*, (*erm*(T)^c^, *cadDX*)	*sec*, (*scn, chp*)		
7		MSSP-ST21-IX	H7a	+					1	C3917	*blaZ*	*lukS/F-I, siet*, *se-int*, *expA*		
	MSSA-ST30-I		H7b	+	+	+	+	+	5	C3916	*blaZ*	*tst*, [*seg, sei, sem, sen, seu*], *sea*, (*chp, sak*)		
	MSSA-ST5-J		H7c	+	+				2	C3918	*blaZ*, *erm*(C)^c^	*lukED*, (*scn, chp, sak*)		
Total			23	17	17	16	17	14	81					

MSSA, methicillin-susceptible *S. aureus*; MSSP, methicillin-susceptible *S. pseudintermedius*.

a Clones were designated as follows: bacterial species (MSSA or MSSP)-MLST-PFGE profile.

b Genes physically linked are displayed in square brackets and those possibly linked based on bibliography are shown in brackets.

c Inducible clindamycin resistance.

**Table 2:** Different *spa* types present among the *S. aureus* clones per household during the sample period (T0–T4), positive individuals and correspondent clones.

Case	*spa*	repeats	Clone	Sampling	Individual/s
1	t021	15-12-16-02-16-02-25-17-24	MSSA-ST1654-A	T0, T1, T2, T3, T4	H1a, D1a^T0^
	t159	14-44-13-12-17-17-23-18-17	MSSA-ST2619-B	T3	D1a
2	t159	14-44-13-12-17-17-23-18-17	MSSA-ST121-C	T0, T1, T2, T3, T4	H2a, H2b^T1^, H2c^T1^, D2a^T0^
	t002	26-23-17-34-17-20-17-12-17-16	MSSA-ST5-D	T0, T1	H2d, H2e^T0^
	t1451	08-16-02-25-34-25	MSSA-ST398-E	T3, T4	H2b, H2d
3	t073	**08-16-02-16-13- -17-34-16-34**	MSSA-ST45-F	T0, T1, T2, T3, T4	H3a_1_^T0, T3, T4^, H3a_2_, D3a
	t116	**08-16-02-16-13-13-17-34-16-34**	MSSA-ST45-F	T1	H3a_1_
	t026	**08-16- - - - - - - -34**	MSSA-ST45-F	T2	H3a_1_
4	t151	26-17-20-17-16	MSSA-ST121-G	T0	D4a
6	t1071	26-23-12-23-02-34-34	MSSA-ST30-H	T1, T2, T3, T4	H6b
	t1451	08-16-02-25-34-25	MSSA-ST398-E	T3	D6b
7	t012	15-12-16-02-16-02-25-17-24-24	MSSA-ST30-I	T0, T1, T2, T3, T4	H7b
	t002	26-23-17-34-17-20-17-12-17-16	MSSA-ST5-J	T0, T1, T2	H7_C_

When an individual was not positive for the specific *spa* type in all indicated samplings the positive sampling is indicated in superscript. Boldface and dashes added to facilitate comparison of *spa* repeats in the different isolates of clone MSSA-ST45-F.

### SP Isolates and Clones Recovered and SP Population Structure

Eight SP isolates were recovered from 16 owners and 18 isolates from 10 dogs. Including the 5 SP isolates obtained in the former report (T0) [7], a total of 31 SP isolates were recovered from the 7 cases.

Based on their PFGE band profile, 9 SP clones and 9 distinct STs (ST71, ST77, ST6, ST42, ST142, ST185, ST100, ST70 and ST21) were identified (Table 1). Four subclones (VIIIa to VIIId) of MSSP-ST70-VIII were detected (Table 1).

### Antimicrobial Resistance Profile of SA and SP Isolates

All SA isolates were susceptible to oxacillin and cefoxitin and were *mec*A negative (Table 1). All SA isolates from the same clone presented identical antimicrobial resistance phenotypes. None of the SA clones were multidrug resistant (MDR) (resistance to at least three classes of antimicrobials). The majority of the SA clones (6/10) was susceptible to all antimicrobials or showed only penicillin resistance harboring the *blaZ* gene. Two clones (MSSA-ST398-E, MSSA-ST5-J) presented additional MLS_B_ resistance [*erm*(C) or *erm*(T)], and a single clone (MSSA-ST121-C) tetracycline resistance [*tet*(K)].

All SP isolates from the same clone presented identical antimicrobial resistance phenotype. A single sporadic MRSP clone (MRSP-ST71-I) and 2 MSSP (MSSP-ST77-II, MSSP-ST142-V) were MDR (Table 1). The remaining SP clones obtained were susceptible to all antimicrobials or showed only penicillin resistance (*blaZ*).

### Presence of Virulence Genes in SA and SP

Virulence traits of SA and SP clones are shown in Table 1. All isolates lacked the PVL-toxin genes *lukS/F-PV*. All SA representative strains (one isolate per clone, per household) but one carried enterotoxin genes, with 63.6% of them harboring different enterotoxin-gene-cluster combinations. Furthermore, 45.5% of them exhibited the leukocidin *lukE/D* genes and 18.2% of clones the exfoliatin gene *etb*. All SP representative isolates tested harbored the *lukS/F-I*, *siet* and *se-int* and 3 (33.3%) the *expA* gene.

### SA and SP Species Distribution along the Sample Year

**Figure 2:**
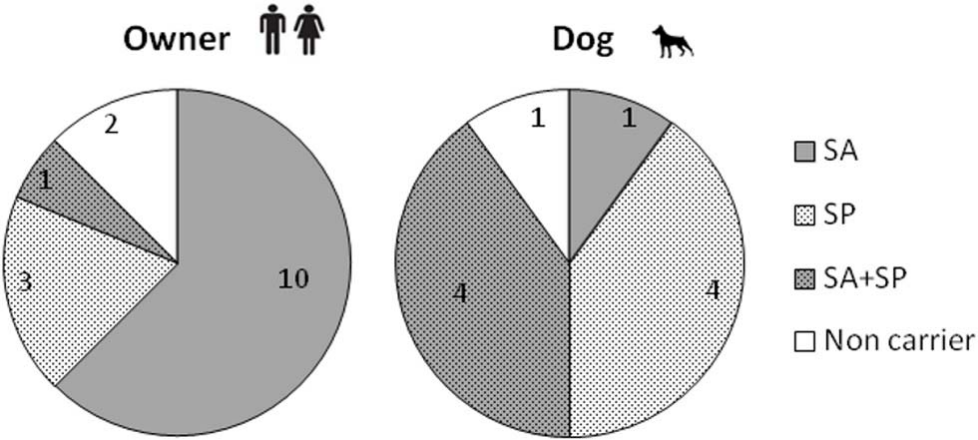
Distribution of investigated individuals (owners, dogs) related to the SA and/or SP carriage as a summary of the sampling year (T0–T4).

Most positive owners (10/14) carried only SA throughout the sampling year. However, three owners only exhibited SP (Figure 2). Both, SA and SP were obtained along the study in only one person. In contrast, both bacterial species were recovered from a high number of positive dogs (4/9) along the study, with a single dog carrying only SA. Four animals were only positive for SP.

### SA and SP Carriage Status

**Figure 3:**
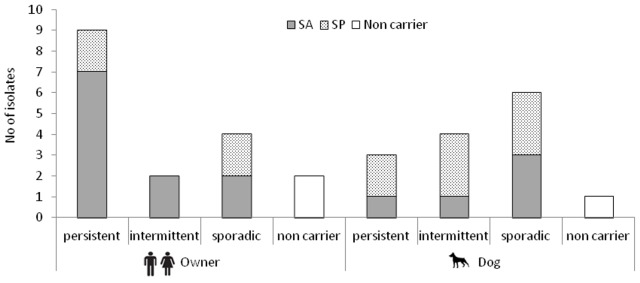
Number of investigated individuals (owners and dogs) with different *S. aureus* (SA) and *S. pseudintermedius* (SP) carriage status over the sample year (T0–T4). It should be noted that the sum is higher than the total number of isolates given that individuals could belong to 2 distinct carriage types depending on bacterial species.

The carriage status of the investigated subjects over time is shown in Figure 3. Among the 16 owners, seven were persistent carriers of SA and 2 of SP. None of these individuals presented SA or SP co-carriage along the sampling period (Figure 1). Two humans were intermittent SA carriers. Two owners were sporadic SA carriers and 2 sporadic SP carriers. A single owner (H2b) was carrier of both bacterial species along the year (intermittent SA carrier and sporadic SP carrier) (Figure 1). Only 2 owners were non-carrier throughout the sample year.

The majority of dogs (6/10) were sporadic nasal carriers of SP (n = 3) or SA (n = 3). Three dogs were intermittent SP carriers and one dog was a SA intermittent carrier, with 2 of these animals being also sporadic SA carriers. Two animals were SP persistent carriers and one was a persistent carrier of SA. Among these, two dogs were additional sporadic SA and SP co-carriers (Figure 1). Remarkably, 40% of dogs belonged to 2 distinct carriage types depending on bacterial species. A single dog was non-carrier.

### Dynamics of the Interspecies Transmission Cases over Time

#### Cases of direct SA transmission

All three households with presumed SA direct transmission (cases 1, 2 and 3) maintained the original SA clone in the owners implicated in the index cases while only one index dog (D3a) was a persistent carrier (MSSA-ST45-F) (Table 1, Figure 1).

In case 2, index dog D2a revealed to be a persistent SP carrier due to the presence of a MDR SP clone (MSSP-ST77-II) in samplings T1, T2 and T3 (Table 1, Figure 1). A coexisting owner (H2b) harbored the aforementioned SP clone in sampling T1, what represents a novel sporadic case of direct SP interspecies transmission. It is interesting to underline the presence of a SA t1451 clone (MSSA-ST398-E) that belongs to the lineage ST398 in two owners (H2b and H2d) in two consecutive samplings (T3–T4).

#### Indirect SA anthropozoonotic transmission

The dog from case 4 (D4a), positive for a MSSA-ST121-G isolate only in the initial sampling, was positive for a SP clone (MSSP-ST42-IV) in the three following samplings (Table 1, Figure 1), while its owner was a non SA or SP carrier.

#### Direct SP transmission

In case 5, owner and dog (H5a and D5a) were persistent SP carriers. In the last 3 samplings (T2 to T4) a different SP clone (MSSP-ST185-V) from the index case was observed. (Table 1, Figure 1).

#### Cases of indirect SP zoonotic transmission

As for case 6, the index human (H6a) was permanently colonized by SP, with MSSP-ST100-VII isolated in T0, and MSSP-ST70-VIII in the subsequent samplings (T1 to T4) (Table 1, Figure 1). Interestingly, both STs only differed in a silent mutation in allele *tuf* (*tuf*_2 at position T136A) and presented the exfoliatin gene *expA* (Table 1). The coexisting human (H6b) was a persistent carrier of a SA clone (MSSA-ST30-H). Remarkably, one dog (D6b) carried a MSSA t1451-ST398 isolate in one sampling (T3).

In case 7, neither the index human (H7a) nor any of the coexisting individuals carried the original MSSP ST21 clone in subsequent samplings (Table 1, Figure 1).

### Co-carriage with MRCoNS and Molecular Characterization of Isolates

Thirty-two MRCoNS isolates (28 *S. epidermidis*, 3 *S. haemolyticus* and 1 *S. succinus*) were detected along the four samplings from a high rate of owners (14/16) and in half of tested dogs (5/10), of which 19 (18 *S. epidermidis* and one *S. haemolyticus*) were present with SA or SP and further investigated (Figure 1). In addition to the *S. haemolyticus* clone (MRSH-6), 9 MRSE clones were observed by *Sma*I-PFGE, with clone 4 presenting two subclones (4a–4b) (Table 3).

**Table 3:** Characteristics of the MRCoNS recovered in the SA/SP positive individuals per household.

						Antimicrobial resistance profile	Concomitant SA/SP clone	
Case	Clone	Individual	Sampling	Isolate	Phenotype of resistance^a^	Resistance genes detected	SA	SP
1	MRSE-1	D1a	T3	C4812	P-O-F-E	*blaZ, mec*A, *mphC, msrA/msrB*	MSSA-ST2619-B	
2	MRSE-2	H2a	T2	C4807	P-O-F-E-M	*blaZ, mec*A, *mphC, msrA/msrB, mupA*	MSSA-ST121-C	
	MRSE-3	H2d	T4	C5588	P-O-F	*blaZ, mec*A	MSSA-ST398-E	
3	MRSE-4a	H3a_1_	T1	C3956	P-O-F	*blaZ, mec*A	MSSA-ST45-F	
	MRSE-4b	H3a_1_	T3	C5097	P-O-F-M	*blaZ, mec*A, *mupA*	MSSA-ST45-F	
	MRSE-4b	H3a_2_	T2	C4798	P-O-F-M	*blaZ, mec*A, *mupA*	MSSA-ST45-F	
	MRSE-5	H3a_2_	T1	C3958	P-O-F-T-E-To	*blaZ, mec*A, *tet*(K), *tet*(M), *mphC, msrA/msrB, aadD*	MSSA-ST45-F	
	MRSE-5	H3a_1_	T2	C4796	P-O-F-T-E-To	*blaZ, mec*A, *tet*(K), *tet*(M), *mphC, msrA/msrB, aadD*	MSSA-ST45-F	
	MRSE-5	H3a_1_	T4	C5573	P-O-F-T-E-To	*blaZ, mec*A, *tet*(K), *tet*(M), *mphC, msrA/msrB, aadD*	MSSA-ST45-F	
5	MRSH-6	D5a	T1	C3960	P-O-F-E-X-Fu^b^	*blaZ, mec*A, *mphC, msrA/msrB, dfr*(A), *dfr*(G)		MSSP-ST142-V
	MRSE-7	D5a	T4	C5577	P-O-F	*blaZ, mec*A		MSSP-ST185-VI
6	MRSE-8	H6a	T1	C3945	P-O-F-G-K-M-Fu^b^	*blaZ*, *mec*A, *aacA/aphD, mupA*		MSSP-ST70-VIIIa
	MRSE-8	H6a	T2	C4791	P-O-F-G-K-M-Fu^b^	*blaZ*, *mec*A, *aacA/aphD, mupA*		MSSP-ST70-VIIIb
	MRSE-8	H6a	T3	C5093	P-O-F-G-K-M-Fu^b^	*blaZ*, *mec*A, *aacA/aphD, mupA*		MSSP-ST70-VIIIb
	MRSE-8	H6a	T4	C5564^c^	P-O-F-G-K-Fu^b^	*blaZ*, *mec*A, *aacA/aphD, mupA*		MSSP-ST70-VIIIc
	MRSE-8	H6b	T4	C5558	P-O-F-G-K-M-Fu^b^	*blaZ*, *mec*A, *aacA/aphD, mupA*		MSSP-ST70-VIIIc
	MRSE-9	H6b	T2	C4789	P-O-F-G-To-K	*mec*A, *aacA/aphD*	MSSA-ST30-H	
	MRSE-9	D6c	T2	C4787	P-O-F-T	*blaZ, mec*A, *tet*(K), *tet*(M)		MSSP-ST70-VIIIb
7	MRSE-10	H7b	T4	C5581	P-O-F-E	*blaZ, mec*A, *mphC*, *msrA/msrB*	MSSA-ST30-J	

MRSE, methicillin-resistant *S. epidermidis*; MRSH, methicillin-resistant *S. haemolyticus.*

a P, penicillin; O, oxacillin; F, cefoxitin; E, erythromycin; M, mupirocin; Cp, ciprofloxacin; To, tobramycin; X, co-trimoxazol; Fu, fusidic acid; G, gentamicin; K, kanamycin; T, tetracycline.

b Fusidic acid resistance genes *fusB* and *fusC* were not detected.

c isolate C5564 was mupirocin susceptible regardless the presence of the *mupA* gene.

A total of 15.6% (10/64) of SA/SP positive samples also carried a MDR MRCoNS isolate. Half of MRCoNS clones were MDR carrying a wide variety of antimicrobial resistance genes (Table 3). Interestingly, several isolates of the same clones from cases 3 (MRSE-4) and 6 (MRSE-8) exhibited different antimicrobial resistance pheno- and/or genotypes to mupirocin over time (Table 3). Moreover, clone MRSE-9 in 2 individuals (H6b and D6c) presented different antimicrobial resistance profiles at the same sampling (T2). The trimethoprim resistance *dfr*(G) gene was detected in clone MRSH-6 and MSSP-ST142-V in the same sampling (Table 3).

Half of SA or SP positive owners and 3 of the 9 positive dogs co-carried a MRCoNS isolate in at least one sampling. Among these, two owners (H3a_1_ and H6a) were persistent carriers of both types of staphylococci (Figure 1, Table 3). In case 6, owner H6b and dog D6c harbored the identical MRSE-9 in sampling T2, representing a novel case of direct interspecies transmission, but this time for by MRCoNS isolates.

### Particular Traits of the SA ST398 Isolates

Four SA t1451-ST398 isolates from two owners (H2b, H2d) at successive samplings (case 2) and one from an unrelated household (case 6) from a dog (D6b), belonged the same SA clone (MSSA-ST398-E) based on all molecular techniques performed (Table 1).

Only these isolates exhibited inducible MLS_B_ resistance due to the presence of the *erm*(T) gene. PCR analysis for the presence of the cadmium resistance operon *cadDX* (recently described in physical linkage to *erm*(T) in a small MSSA ST398 plasmid, pUR3912 [43]) revealed their existence in the five isolates. All 5 isolates were tested for virulence determinants and evidenced the presence of the *scn*, *chp* and the enterotoxin gene *sec*. Both positive owners live in an urban area with not contact to livestock or other farm animals. The positive dog lives in a rural area without direct contact to livestock.

## Discussion

This is the first study on the dynamics of colonization of SA and SP in healthy pet-owning household members. All investigated households presented at least one individual positive for SA or SP in each of the samplings. In addition, the vast majority of investigated owners (14/16) and dogs (9/10) carried either SA or SP in at least one sampling, what evidences a real flow of both bacterial species within pet-owning household settings.

Based on the dynamics of colonization of SA it seems that the original source of SA was the human and that dogs serve as sporadic sources of human carriage, which may play a role in re-colonization or maintenance. The household with an identical SA clone in owners and dog along the whole year (case 3) might be explained by the persistent carriage of both index persons. Interestingly, in this household, SA isolates (clone MSSA-ST45-F) with three different *spa* types in serial samplings were recovered from one of the owners (H3a_1_). The identical PFGE band profile in these SA isolates suggests evolution of its *spa* repeat composition rather than the presence of different clones. In this line, mutations over time within the same SA clone have been previously detected in SA longitudinal studies [45]–[48].

The fact that SP was maintained along the whole sample year in two owners reveals that humans can either be carriers for prolonged periods of time of this bacterial species or present easiness for recurrent acquisition. This observation suggests that the transmission capacities of SP, as well as its survival ability on non-natural hosts, may have been overlooked. With this respect, owner and dog from case 5 lived separately for several months between T0 and T1. The absence of SP in the owner in T1 suggests that the source of SP was the dog.

The presence of SP persistent owners, together with the absence of intermittent SP human carriers might point to favourable conditions in specific individuals to be colonized by SP. In contrast, both bacterial species were present in all carriage types in dogs, with SA being in most cases sporadically detected. This observation reflects the ability of dogs to be secondary reservoirs for staphylococcal propagation.

SA and SP carriage evolution showed different trends depending on the host from which they were recovered: (i) a single owner was positive for both bacterial species, what contrasts with the elevated number of positive dogs from which SA and SP was recovered (4/9), and (ii) SP was not detected in the SA-persistent owners, and SA was not present in the SP-persistent humans, while two of the three persistently colonized dogs were sporadic nasal carriers of the other coagulase positive staphylococci. These observations suggest that these bacteria might present a host-dependent behaviour and/or that they may interfere with each other’s colonization in the human host.

While SA normally colonizes the nasal cavity of humans [1], SP seems to be more variably located in dogs, and perineum and mouth have been recently addressed as the body-sites with most SP recovery rates [2], [49]. A recent study focused on the dynamics of SP in healthy dogs with multiple sampling sites, revealed that between 2 and 5 clones were present in the same animal, what is in agreement with our results. Replacement of SP clones in an individual microbiota over time has been previously reported as common in previous longitudinal studies in SP in dogs [50]. In contrast, the SA population structure was mostly maintained in the SA persistent individuals, what is in line with other longitudinal SA studies on humans [16]. To our knowledge, no information is available on preferential colonization sites of SA in dogs and SP in humans, what warrants further investigations.

MSSA strains belonging to the same genetic background as pandemic MRSA clones (CC45, CC121, CC30, CC398, CC5) were collected from humans, of which CC121 and CC398 were also sporadically detected in dogs. The elevated virulence gene content of all SA clones detected enhances the importance to control possible sources for acquisition of potential pathogenic bacteria. In addition, all SA clones but one, detected in a dog (MSSA-ST121-G), harbored genes implicated in evasion of the human immune system, what responds to a human adaptation.

Interestingly, all 5 MSSA-ST398-E isolates (4 from 2 humans, 1 from dog) carried the MLS_B_ resistance gene *erm*(T) and the cadmium resistance operon *cadDX*, which has been recently described to be co-located within pUR3912 [44]. The ability of this small plasmid to integrate within the chromosomal DNA [51] suggests a plausible clonal spread of pUR3912 among animal-independent MSSA ST398 isolates. Positive owners did not have direct contact to rural areas or farm animals and the presence of the IEC genes points to a human adaptation [52]. The detection of an enterotoxin gene (*sec*) in all 5 isolates is remarkable, given that these toxin genes are normally absent among MRSA ST398 isolates. The presence of the same *Apa*I-PFGE band profile between unrelated individuals suggests a clonal distribution.

Co-carriage of SA and MRCoNS has been previously reported to have a negative association due to competition for the same ecological niche [53], [54]. Moreover, *S. epidermidis* is considered to “protect” the host from SA colonization [54]. Half of SA or SP positive owners also presented MRCoNS in at least one sampling, what reflects a conceivable punctual coexistence of such bacteria. Further, the persistent co-carriage of MRSE and SA or SP observed in two owners (12.5% of investigated humans) may represent a source of antimicrobial resistance genes for SA and SP.

The detection of a MRSE sporadic case of interspecies transmission (which direction remains unknown) that involved isolates with different antimicrobial resistance profiles evidences transmissibility potential. The presence of mupirocin resistance *mupA* gene in 42% (8/19) of MRCoNS isolates represents a potential risk for acquisition during SA/SP intended decolonization with mupirocin-based treatments. Further, the differential evolution of mupirocin resistance in some isolates of the same clone deserves continued surveillance. Although the acceptance of exogenous genetic material between staphylococci does seem to require special circumstances not yet properly understood [31], transmission of the *mec*A gene between MRCoNS and SA has been previously assumed [28], [29], [31].

### Conclusions

This study gains knowledge in the ecology of SA, SP and MRCoNS in humans and in-contact dogs and represents a first approach to understand the role that dogs may play in the epidemiology of SA and SP in the human host over time. The apparent adaptability of SP to colonize dog owners who do not carry SA, as well as great predisposition of these animals to be SA carriers should be taken into account in subsequent developments of infection control measurements. Moreover, the human-associated MSSA ST398 is shown to be transferred and maintained within household members. Further longitudinal studies on SA and SP with a larger cohort in humans and in-contact dogs are essential for confident assertions of the implication of pet-owning as increased risk factor to acquire and maintain potentially pathogenic bacteria.
