# Correction: Clonal Dynamics of Nasal *Staphylococcus aureus* and *Staphylococcus pseudintermedius* in Dog-Owning Household Members. Detection of MSSA ST^398^

**DOI:** 10.1371/annotation/8f8893cc-1367-410d-a450-d72a19a56643

**Published:** 2013-12-20

**Authors:** Elena Gómez-Sanz, Carmen Torres, Sara Ceballos, Carmen Lozano, Myriam Zarazaga

A funding organization was incorrectly omitted from the Funding Statement. The Funding Statement should read: "This study was financially supported by Projects SAF2009-08570 and SAF2012-35474 from the Ministerio de Economía y Competitividad of Spain and Fondo Europeo de Desarrollo Regional (FEDER). EGS has a fellowship from the Gobierno de La Rioja of Spain, and CL has a fellowship from the Ministerio de Economía y Competitividad of Spain. The funders had no role in study design, data collection and analysis, decision to publish, or preparation of the manuscript." 

